# Global Analysis of a Key Developmental Pathway in Plants

**DOI:** 10.1371/journal.pbio.0040172

**Published:** 2006-05-02

**Authors:** Liza Gross

Stem cells are most often associated with mammalian development, but plants have them too. With a modular architecture that allows ongoing replacement of new stems, leaves, roots, and flowers, plants escape the debilitating effects of aging that other multicellular organisms endure. The raw materials for regeneration come from reserves of stem cells—sequestered in two meristems, one for shoots and another for roots—that adult plants draw on throughout their lives.

The root meristem consists of the quiescent center (QC)—a group of four cells that maintain neighboring cells as stem cells—and the undifferentiated “initial cells” that give rise to the concentrically organized cylindrical root tissues: the epidermis, ground tissue (made up of the cortex and endodermis), and stele (pericycle and vascular cylinder). Initial cell divisions are asymmetrical, resulting in one renewed initial cell and a daughter cell that differentiates. A key regulator of root development, the transcription factor SHORT-ROOT (SHR) acts in the QC and endodermis to regulate stem-cell specification and radial patterning.

The genetic workhorse of plant biology *Arabidopsis thaliana* has shed considerable light on the molecular pathways controlling these processes, yet many details remain obscure. In a new study, Mitch Levesque, Teva Vernoux, Philip Benfey, and colleagues focused on SHR to better understand its role in root development and identify those genes that are directly controlled by it. Before their study, only one of SHR's gene targets, *SCARECROW (SCR)*, had been identified. The researchers also demonstrate the value of applying meta-analysis—a standard statistical approach used in many other fields to integrate and interpret the results of multiple independent studies—to the analysis of transcriptional networks in development.

To identify the targets of a transcription factor, researchers typically alter their activity and then analyze genome-wide transcription with microarray analysis—an approach that proves cumbersome in multicellular organisms, where genes are often expressed in different cells at different times. Meta-analysis can overcome this problem, Levesque et al. argue, because it can detect subtle patterns in larger datasets that might be overlooked in smaller ones. Using this approach, the researchers identified eight direct SHR transcriptional targets, including *SCR*, as well as a long list of indirect targets involved in cell signaling and hormonal responses. They also revealed a new function for the transcription factor.

First, they constructed a form of SHR that allowed them to exert precise control over its temporal and spatial expression by administering a synthetic hormone called dexamethasone (Dex). Plants lacking *SHR* ( *shr-2* mutants) have much shorter roots and defective radial patterning. But *shr-2* mutants bred to express the SHR construct had normal root growth, which the researchers attribute to restored stem-cell activity. By crossing this strain with another engineered to express a fluorescent protein upon *SCR* transcription, the researchers could predict when SHR targets were expressed after Dex treatment. Then, adding a compound (called cycloheximide) expected to block expression of genes that act further downstream in a pathway, they demonstrated that SHR directly targets *SCR*.

To identify other direct SHR targets, the researchers altered SHR activity in their transgenic *shr-2*:SHR mutants using three different experimental treatments, then collected transcriptional profiles from the root tips of five-day-old plants. The meta-analysis of the three microarray datasets identified eight candidate targets—four of which can bind to SHR in plant cells. Which is not to say that SHR can't bind to the other four genes, only that these assays did not provide that evidence. Future studies can resolve this question.

All eight candidate targets were expressed to some degree in cell types expressing SHR. Three target genes were expressed in stele tissues, the root's vascular tissue. Levesque et al. found that the stele was narrower and had fewer initial cells in *shr-2* mutants, demonstrating a new role for SHR in stele differentiation and development.

The researchers go on to show that hundreds of indirect target genes are either activated or repressed in response to SHR activity and that many of these genes are involved in cell or hormonal signaling pathways. They plan to investigate the functional significance of these genes in future experiments.

Overall, these results suggest that SHR directly activates *SCR*, and in doing so influences QC specification and asymmetric cell division. It does not work alone, however, since simply adding *SCR* to *shr-2* mutants did not correct defects associated with these processes. SHR operates in at least five regions, Levesque et al. conclude: the QC, early and late endodermis, and early and late stele. SHR controls root development, they propose, by coordinating overlapping transcription, signaling, and hormonal pathways. The product of these interactions determines how SHR influences stem-cell niche specification, radial patterning, and stele development. Functional analysis of the different targets will help the researchers test the validity of their model.

## 

**Figure:**
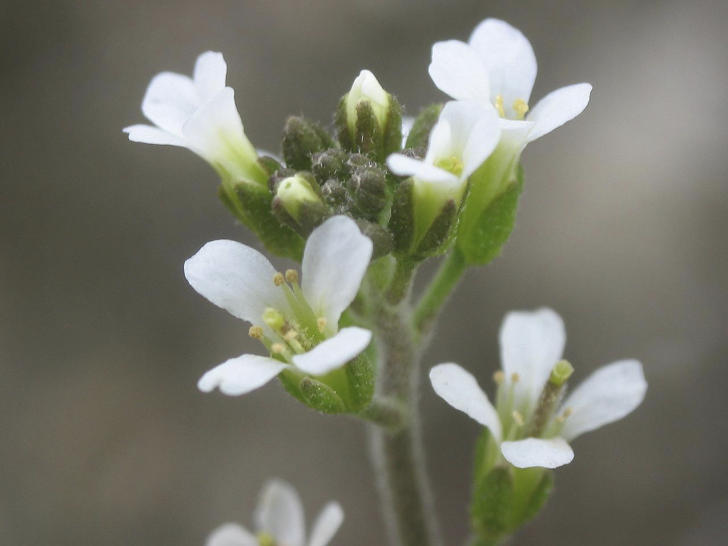
Arabidopsis thaliana

